# Prenatal and Peripartum Management of Patients with Hypofibrinogenemia Resulted in Two Successful Deliveries

**DOI:** 10.1155/2017/9427359

**Published:** 2017-02-14

**Authors:** Yuko Teraoka, Hiroshi Miyoshi, Kumi Oshima, Satoshi Urabe, Norifumi Tanaka, Yoshiki Kudo

**Affiliations:** ^1^Department of Obstetrics and Gynecology, Graduate School of Biomedical Science and Health Sciences, Hiroshima University, Hiroshima, Japan; ^2^Department of Hematology and Oncology, Research Institute for Radiation Biology and Medicine, Hiroshima University, Hiroshima, Japan

## Abstract

Fibrinogen is an essential agent involved in maintaining pregnancy and coagulation. Since inherited fibrinogen disorders introduce greater risks for conditions such as placental abruption and postpartum hemorrhage, careful prenatal and perinatal management is essential for this patient population. We report two cases of successful deliveries in patients with hypofibrinogenemia. Case 1 is of a 26-year-old (gravida 1, para 1) woman. The patient's fibrinogen level increased spontaneously to higher than 300 mg/dL during pregnancy, without treatment. She delivered at week 38 of gestation, with no complications. Case 2 is of a 30-year-old (gravida 3, para 1) woman. We performed repeated infusions of fibrinogen to maintain the level higher than 100 mg/dL during pregnancy and at least 200 mg/dL in the perioperative period; the patient delivered a healthy infant. We identified a new mutation, Hiroshima I (*γ*278Tyr→His). It is important to maintain appropriate fibrinogen levels in cases of inherited fibrinogen disorders for successful prenatal and peripartum management.

## 1. Introduction

Fibrinogen is a 340 Da glycoprotein synthesized in the liver, which is involved not only in coagulation but also in maintaining pregnancy. Inherited fibrinogen disorders are divided into two different types: quantitative defects, that is, afibrinogenemia or hypofibrinogenemia, and qualitative defects, that is, dysfibrinogenemia. The prevalence of afibrinogenemia is about 1 in 1 million [1]. Mutations related to these disorders have been detected all over the world. The patient with afibrinogenemia is usually identified at birth by the presence of bleeding from the umbilical cord. However, hypofibrinogenemia and dysfibrinogenemia are usually asymptomatic. Appropriate control of the fibrinogen level is necessary for successful prenatal and peripartum management. It is necessary to maintain the fibrinogen level higher than 100 mg/dL during pregnancy [2, 3]. After the onset of labor, the fibrinogen level must be maintained to higher than 150 mg/dL or 200 mg/dL in case of caesarean section [2, 3]. Here, we report two cases of hypofibrinogenemia with different course during pregnancy.

## 2. Case Report

*Case  1*. A 26-year-old (gravida 1, para 1) woman presented with a positive pregnancy test result. She had been diagnosed with hypofibrinogenemia at the age of 23 during blood testing to be a bone-marrow transplantation donor. Her personal and family histories were negative for hemorrhagic or thrombotic episodes. At 25 years of age, the patient delivered a girl at 39 weeks of gestation. Blood tests were performed monthly to check the fibrinogen levels. Since her fibrinogen level increased spontaneously during pregnancy, she did not receive infusion therapy. Her prepregnancy fibrinogen level was 76 mg/dL. During the first trimester, it increased gradually to 119 mg/dL and was more than 300 mg/dL in the third trimester. She was admitted to our hospital with rupture of the membranes at 38 weeks and 2 days' gestation. Her coagulation tests showed a normal prothrombin time (PT) of 12.2 s (normal: 10–13 s), activated partial thromboplastin time (APTT) of 28.0 s (normal: 25–38 s), and fibrinogen concentration of 311 mg/dL (normal: 200–400 mg/dL). After inducing labor with prostaglandin E_2_, a 3206 g healthy male infant was delivered. His fibrinogen level was 171 mg/dL. Though the total amount of bleeding was 828 mL because of atonic bleeding, uterine contraction improved with intravenous oxytocin injection and no complications occurred during puerperium.

*Case  2*. A 30-year-old (gravida 3, para 1) woman presented with a positive pregnancy test result. She was diagnosed with hypofibrinogenemia at the age of 25 following an emergency caesarean section with her first pregnancy. She was admitted to the previous hospital to induce labor at 40 weeks and 2 days' gestation, and although there were no complications during labor initially, the patient suddenly developed abnormal vaginal bleeding. Although an emergency caesarean section was performed under general anesthesia, because of suspected placental abruption, no surgical and pathological findings were found to confirm this suspicion. The female infant was healthy with a birth weight of 2120 g. Postpartum hemorrhage (PPH) occurred and the fibrinogen level was 60 mg/dL postoperatively. Immediate transfusion therapy using fresh-frozen plasma (FFP) was administered; this was the only abnormal bleeding episode experienced by the patient. The infant's fibrinogen level was also low, and inherited hypofibrinogenemia was suspected. The patient's history included an artificial abortion at the age of 21 and a spontaneous abortion at 29. She underwent a dilatation and curettage in these two pregnancies at 8 weeks' gestation, with no fibrinogen infusion. She underwent preoperative coagulation tests, only PT and APTT, the first time, and her fibrinogen level was monitored in the perioperative period after she was diagnosed with hypofibrinogenemia the second time. Her fibrinogen level was 66 mg/dL. The surgeries were completed with no complications. The patient's mother received red blood cell (RBC) infusion therapy after delivery; however, there was no other family history of coagulation testing. The patient's two brothers and their families' histories were negative for hemorrhagic and thrombotic episodes. The patient first visited our hospital at 6 weeks' gestation during this pregnancy. Her coagulation tests showed PT of 12.5 s, APTT of 40.1 s, and fibrinogen concentration of 75.9 mg/dL (normal: 200–400 mg/dL). Hematology doctors were consulted, and fibrinogen infusion therapy was started at 7 weeks' gestation. The patient's fibrinogen level was monitored weekly during her pregnancy. She received 3 g of fibrinogen weekly to maintain a fibrinogen level higher than 100 mg/dL [2, 3]. Beginning in the third trimester, physiological elevation of fibrinogen allowed extension of the infusion therapy interval to every three weeks. Fetal growth slowed at 27 weeks of gestation, and a diagnosis of fetal growth restriction (FGR) was made. The patient was hospitalized at 31 weeks' gestation to monitor FGR, because the fetal growth worsened: the estimated fetal weight (EFW) below −2.5 Standard Deviation (SD) from the population standard. However, the fetus grew at the same rate (−2.5 SD) as the fetal growth curve during hospitalization. Doppler ultrasound of fetal blood vessels was normal. A selective caesarean section was performed at 36 weeks and 2 days' gestation under general anesthesia to avoid the risk of bleeding on the recommendation of the anesthesiologist [4]. The male infant had Apgar scores of 8 and 10 at 1 and 5 min, respectively, with a birth weight of 1724 g. The patient received 3 g of fibrinogen for 3 consecutive days, 2 days before the surgery, to maintain the fibrinogen level higher than 200 mg/dL in the perioperative period. The fibrinogen level was 228 mg/dL on the day of operation. Total bleeding during the operation was 260 mL. The placenta was hypoplastic, with a weight of 350 g and marginal insertion of the cord. Since fibrinogen level of the infant persisted at 40–50 mg/dL, he was also suspected to have inherited hypofibrinogenemia, as was the case with his sister. The postoperative course was uneventful. One month after delivery, the patient's fibrinogen level decreased to 68 mg/dL.

We were unable to get patient consent in case 1; therefore, genetic analysis was only conducted in case 2. Her functional fibrinogen level was 112.0 mg/dL and immunological level was 115.7 mg/dL; therefore, congenital hypofibrinogenemia was suspected. A new mutation (*γ*278Tyr→His) was identified as leading to hypofibrinogenemia. Inherited fibrinogen disorders are named after the city where the patient lives or after the city in which the patient was evaluated. Therefore, this case was named “Hiroshima I.” The same mutation was also detected in her two children.

## 3. Discussion

Inherited fibrinogen disorders are very rare; the number of certified patients was 70 according to the national survey on coagulation disorders 2015 [4]. Inherited fibrinogen disorders are divided into two different types: quantitative defects (i.e., afibrinogenemia or hypofibrinogenemia) and qualitative defects (i.e., dysfibrinogenemia) (Table 1). No complications were observed during the pregnancy in case 1, whereas abnormal bleeding occurred after the caesarean section in case 2. Coagulation tests for congenital hypofibrinogenemia and dysfibrinogenemia are usually normal, with the exception of fibrinogen levels [1]. Two measurement methods (PT-derived method and immunoturbidimetry) are used to distinguish these two conditions. The PT-derived method can detect functional fibrinogen level and immunoturbidimetry can reveal immunologic fibrinogen level [1, 5]. Hypofibrinogenemia shows a low level of fibrinogen in both methods and dysfibrinogenemia only demonstrates low functional assay. The functional and immunological level in case 2 showed a proportional decrease; therefore, congenital hypofibrinogenemia was suspected.

**Figure 1:**
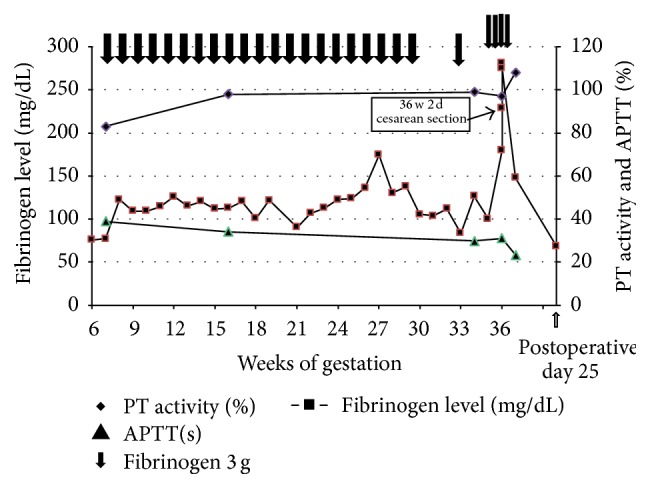
Perinatal course and fibrinogen infusion therapy in case 2.

Fibrinogen also plays an important role in adhesion between the placenta and the uterus; therefore, while it is not essential for implantation, it is necessary for maintaining pregnancy. According to some case reports, pregnancy in the patients with afibrinogenemia will result in abortion without fibrinogen infusion therapy by at least 5 weeks' gestation [2, 3]. Moreover, fibrinogen disorders can cause recurrent abortion, subchorionic hematoma, placental abruption, and PPH [2, 3]. It is known that the minimal fibrinogen level for maintaining pregnancy is above 60 mg/dL [2, 3]. As fibrinogen decreases under threatened premature labor or inflammation, it is necessary to maintain the fibrinogen level higher than 100 mg/dL during pregnancy [2, 3]. After the onset of labor, the fibrinogen level must be maintained to higher than 150 mg/dL or 200 mg/dL in case of caesarean section [2, 3]. The fibrinogen level increased spontaneously to higher than 200 mg/dL during pregnancy in case 1; therefore, she did not receive infusion therapy. In case 2, the patient received 3 g of fibrinogen (half-life of 3-4 days) once a week through the second trimester (Figure 1, Table 2). Beginning in the third trimester, the physiological elevation of fibrinogen allowed extension of the infusion therapy interval to every 3 weeks (Figure 1). The fibrinogen level after surgery stayed higher than 150 mg/dL. Fetal growth restriction occurred, the same as with her previous one. Pathology of the placenta revealed no specific information leading to FGR, such as thrombus or infarction. We did not find out the literature showing relationship between FGR and hypofibrinogenemia and fibrinogen infusion therapy. Marginal insertion of the cord might be one of the reasons for FGR in this case.

Fibrinogen consists of three polypeptide chains (A2*α*, B*β*, and *γ*) encoded by three genes (FGA, FGB, and FGG, resp.) located on the long arm of chromosome 4 [6]. Afibrinogenemia is caused by variations in the FGA, FGB, and FGG genes [7]. Afibrinogenemia is associated with homozygous or compound heterozygous mutation. On the other hand, hypofibrinogenemia is usually related to heterozygous mutation. Mutations causing afibrinogenemia and hypofibrinogenemia in FGA gene are mainly deletions, frameshift, nonsense, or splicing mutations [7]. In contrast, mutations in FBG and FGG include an excess of missense mutation [7]. Genetic analyses have shown the mechanisms regulating the production of fibrinogen. For example, the Matsumoto IV (*γ* 153 Cys→Arg) mutation affects the proximal A region of the *γ* D domain resulting in abnormal folding without intrachain disulphide bond [4, 8]. In case 2, genetic analysis revealed a new mutation (*γ* 278 Tyr→His) named as Hiroshima I on exon 8 in FGG which can affect mRNA splicing or stability or protein synthesis, assembly, and secretion.

We reported two cases of hypofibrinogenemia with different courses during pregnancy. The personal and family history must be checked carefully in case of fibrinogen disorders, as occasionally hereditary abnormalities are found, such as in case 2. For the patients with the fibrinogen disorders, the fibrinogen level must be measured continuously and infusion therapy is essential not only maintaining the pregnancy but also preventing PPH and other complications.

## Figures and Tables

**Table 1:** Characteristics of inherited fibrinogen disorders.

	Afibrinogenemia	Hypofibrinogenemia	Dysfibrinogenemia
Transmission	AR	AD/AR	AD

Fibrinogen level (mg/dL)	<10	70–100	Lower limit of normal

Laboratory	PT-APTT-TT	PT: normal or prolonged	PT-APTT: normal
	Markedly prolonged	APTT: normal	

Symptoms	Umbilical cord bleeding	No symptoms Abnormal bleeding after trauma or operation	No symptoms Abnormal bleeding after trauma or operation Thrombosis
	Recurrent abortion		
	Cutaneous bleeding		
	Intracranial bleeding		
	Gastrointestinal bleeding		
	Hypermenorrhea		

AR; Autosomal recessive, AD; Autosomal dominant, PT; prothrombin time,

APTT; actinated partial thromboplastin time, TT; thrombin time.

**Table 2:** Fibrinogen level during pregnancy in case 2.

Weeks of gestation	Fibrinogen level (mg/dL)
9	109.2
10	109.2
11	114.6
12	125.9
13	115.5
14	120.7
15	112
16	112.9
17	120.7
18	100.6
19	121.8
21	90.5
22	106.8
23	112.9
24	122.8
25	123.9
26	136.5
27	174.4
28	130.5
29	137.8
30	105.5
31	103.7
32	112
33	83.6
34	126.5
35	100.2
36	180.3
Operation day	228.8
POD1	281.4
POD4	274.7
POD7	147.8
POD25	68

POD: postoperative day.
